# A Decade of Post-Intensive Care Syndrome: A Bibliometric Network Analysis

**DOI:** 10.3390/medicina58020170

**Published:** 2022-01-23

**Authors:** Nicolas Paul, Valentina Albrecht, Claudia Denke, Claudia D. Spies, Henning Krampe, Björn Weiss

**Affiliations:** Charité—Universitätsmedizin Berlin, Corporate Member of Freie Universität Berlin and Humboldt-Universität zu Berlin, Department of Anesthesiology and Operative Intensive Care Medicine, Campus Charité Mitte and Campus Virchow-Klinikum, Charitéplatz 1, 10117 Berlin, Germany; nicolas.paul@charite.de (N.P.); valentina.albrecht@charite.de (V.A.); claudia.denke@charite.de (C.D.); henning.krampe@charite.de (H.K.); bjoern.weiss@charite.de (B.W.)

**Keywords:** bibliometric analysis, critical illness, intensive care unit, PICS, post-intensive care syndrome, research collaboration, research output, survivorship

## Abstract

*Background and Objectives:* In 2012, the umbrella term post-intensive care syndrome (PICS) was introduced to capture functional long-term impairments of survivors of critical illness. We present a bibliometric network analysis of the PICS research field. *Materials and Methods:* The Web of Science core database was searched for articles published in 2012 or later using ‘post-intensive care syndrome’ and variant spellings. Using VOSviewer, we computed co-authorship networks of countries, institutions, and authors, as well as keyword co-occurrence networks. We determined each country’s relative research effort and Category Normalized Citation Index over time and analyzed the 100 most-cited articles with respect to article type, country of origin, and publishing journal. *Results:* Our search yielded 379 articles, of which 373 were analyzed. Annual PICS research output increased from 11 (2012) to 95 articles (2020). Most PICS research originates from the US, followed by England, Australia, the Netherlands, and Germany. We found various collaborations between countries, institutions, and authors, with recent collaborative networks of English and Australian institutions. Article keywords cover aspects of cognitive, mental health, and physical impairments, and more recently, COVID-19. Only a few keywords and articles pertained to PICS prevention and treatment. *Conclusions:* Our analysis of Web of Science-indexed PICS articles highlights the stark increase in PICS research output in recent years, primarily originating from US- and Europe-based authors and institutions. Despite the research field’s growth, knowledge gaps with respect to PICS prevention and treatment remain.

## 1. Introduction

Over the last decades, the number of patients admitted to an intensive care unit (ICU) and the capacities in intensive care medicine have been growing continuously [1]. Although the average age and severity of illness have been increasing [2], mortality rates are steadily declining, which has been attributed to advances in technology and a growing evidence base [3]. As a result, we observe a growing cohort of patients surviving their critical illness.

Initially, research in intensive care focused on interventions to improve ICU-centered and short-term outcome measures, such as ICU or hospital mortality [4]. In the 1980s and 1990s, only a few studies explored mortality, quality of life, and functional outcomes beyond ICU discharge [5,6,7,8,9]. In this millennium, however, the intensive care research community acknowledged that mere survival of critical illness comes short of capturing the poor functional outcome of many ICU patients after leaving the hospital, which constitutes a heavy burden to both patients and caregivers [10]. The 2002 Brussels Roundtable identified the need for research on the determinants of long-term wellbeing and on interventions that improve long-term, patient-centered outcomes [4]. Eight years later, at a Society of Critical Care Medicine conference, a nomenclature was developed to conceptualize and organize functional impairments after ICU discharge [11]. Due to the often-overlapping nature of functional post-ICU impairments, the use of the single term post-intensive care syndrome (PICS) was recommended [11]. PICS comprises new or worsening ICU treatment-associated impairments of cognitive functions, mental health (anxiety, depression, and post-traumatic stress disorder (PTSD)), and physical functions [11]. Moreover, it was recognized that not only patients are commonly affected by PICS but also their caregivers, which was described as PICS-F [11]. Similar to the term post-cardiac arrest syndrome [12], agreement on a common PICS terminology should raise awareness for the prevalence of functional impairments after ICU care [11]. The demand for research and awareness for PICS was reiterated at a Society of Critical Care Medicine stakeholder conference in 2012 [13]. Since its introduction, the PICS framework has become well-established and is now the most commonly used terminology to describe post-ICU impairments.

Marking a decade of PICS research, we observe a surging number of publications which pertain to different aspects of the concept, published by various research groups. Network analysis of bibliometric data of publications on PICS can help understand the current and past PICS research agenda and community. As the most apparent form of collaboration [14], co-authorship networks may facilitate understanding of ongoing and past research collaborations on an individual, institutional, and country level [15,16]. Bibliometric analysis may also reveal the most influential articles, journals, and authors, and identify knowledge gaps in the field.

To our knowledge, a science mapping of publications on PICS has not been performed yet. The aim of this study was to conduct a bibliometric network analysis of PICS research. We quantified the annual research output and visualized co-authorship networks on an individual, institutional, and country level, as well as keyword co-occurrence networks over time. We determined the relative quantity and impact of each country’s research output and analyzed the 100 most-cited articles with respect to article type, country of origin, and publishing journal. Results from our analysis may help identify current and past research trends, common collaborations, and knowledge gaps in the PICS research field.

## 2. Materials and Methods

### 2.1. Web of Science Export and Data Cleaning

On 7 September 2021, we searched the Web of Science core database using the search terms ‘post-intensive care syndrome’, ‘post intensive care syndrome’ and ‘postintensive’ care syndrome’ for all fields. We included articles published in 2012 or later without restrictions with respect to article type and language. Full records of article metrics were extracted and imported to Microsoft Excel. We also extracted a citation report that included each article’s annual citations. Based on titles, articles were screened for suitability by one author (VA). In the case of ambiguity, abstracts and full texts were assessed. After discussion with another author (NP), articles were excluded if they did not pertain to ICU patients and/or PICS.

Titles, abstracts, and, in case of ambiguity, full texts were screened by one author (NP) to assign publications to article types. Reports from consensus and stakeholder conferences were considered original work. Based on authors’ addresses, we identified articles’ countries of origin (one article could be assigned to several countries). For all articles published prior to 2018, the annual number of citations for the publication year and the two following years were calculated. To merge various notations of the same author, institution, or keyword, data were cleaned using OpenRefine (version 3.4.1; Google LLC, Mountain View, CA, USA). Bar graphs were created using Prism 9 (version 9.3.1; GraphPad Software LLC, San Diego, CA, USA).

### 2.2. Distance-Based Networks

Co-authorship networks for countries, institutions, and authors as well as keyword co-occurrence networks were computed using VOSviewer (version 1.6.17 for Mac; Leiden University, Leiden, The Netherlands) [17]. VOSviewer networks consist of items (i.e., countries, institutions, authors, or keywords). The closer items are related to each other, the closer they appear in the network. An item’s size is determined by its importance relative to the other items (i.e., the number of publications or keyword occurrences). Direct links between items indicate immediate connections (i.e., a co-authorship or a co-occurrence of keywords). A link’s thickness indicates its strength (i.e., the number of co-authorships or the number of publications where two keywords co-occur). VOSviewer assigns each item to a cluster of related items [16]. Color overlays indicate the average publication year of articles of the respective item [18]. Balancing readability and information in the visualizations, the minimum number of articles was set to three for the country network, to four for the institution and author networks, and to five for the keyword co-occurrence network.

### 2.3. Category Normalized Citation Index and Relative Research Activity

We calculated each country’s median Category Normalized Citation Impact (CNCI) [19] and relative research activity [20] for three time periods: 2012–2014, 2015–2017, and 2018–2021. The CNCI indicates the ratio of an article’s citations and the average citations of articles within the same research field, document type, and year [19]. Hence, a CNCI >1 or <1 indicates above-average or below-average citations per article, respectively. For the CNCI calculation, we defined our sample of PICS research as the research field of reference, and documents were grouped in original research (including protocol papers), reviews, and other articles (editorials, letters, case reports, meeting abstracts, and book reviews).

The relative research activity indicates the ratio between a country’s PICS research output and the average research output across countries that contribute to PICS research in a given time period. Thus, a relative research activity >1 or <1 indicates above-average or below-average research output, respectively.

### 2.4. Analysis of the 100 Most-Cited Articles

We identified the 100 most-cited articles on PICS and analyzed them with respect to article type, country of origin, and publishing journal. Based on the corresponding author’s affiliation, each of the 100 most-cited articles was assigned to a single country of origin. Journal impact factors were drawn from Clarivate Analytics Journal Citation Reports 2020 [21].

## 3. Results

### 3.1. Study Sample

**Figure 1:**
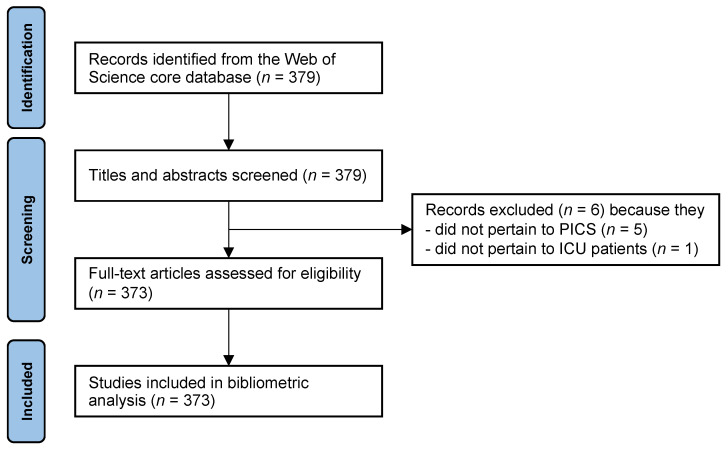
Study inclusion flowchart. ICU, intensive care unit; PICS, post-intensive care syndrome.

The Web of Science search yielded 379 articles, from which six articles were excluded as they did not pertain to ICU patients and/or PICS (Figure 1). Of the remaining 373 articles, 145 were original research articles, 103 reviews, 58 editorial articles or letters, 33 meeting abstracts, 19 protocol papers, nine case reports, five research letters, and one was a book review (Table 1).

### 3.2. Characteristics of Articles

**Figure 2:**
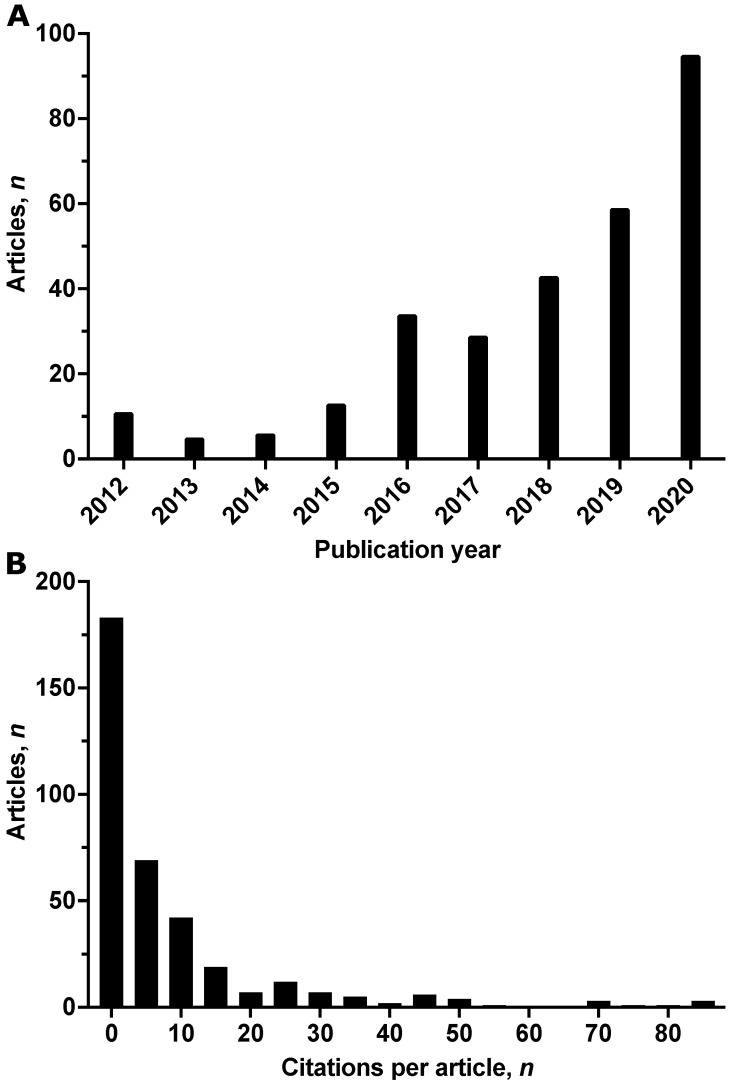
(**A**) Number of articles on post-intensive care syndrome, by year. Up to the Web of Science search on 7 September 2021, 78 articles were published in 2021. (**B**) Histogram of the number of citations per article (bin width of five citations). Eight articles with >100 citations (range: 125–939 citations) are not displayed.

Articles were written by 1621 different authors from 793 institutions and 39 countries, with a mean number of 6.4 (SD 6.3) and a median number of 5 (IQR 3; 8) authors per article. The mean number of countries of origin was 1.4 (SD 1.0) per article, and the median number of countries of origin was 1 (IQR 1; 1) per article. On average, each article had 5.6 (SD 2.5) keywords, with a median of 5 (IQR 4; 6) keywords per article. The annual research output steadily increased from 11 articles in 2012 to 95 articles in 2020 (Figure 2A). Articles were cited 5415 times, with a mean of 14.5 (SD 57.3) citations per article and a median of 3 (IQR 0; 11) citations per article. Eight articles (2%) were cited >100 times, while 116 articles (31%) were uncited, and 67 articles (18%) were cited once or twice (Figure 2B).

### 3.3. Bibliometric Analysis by Country

**Figure 3:**
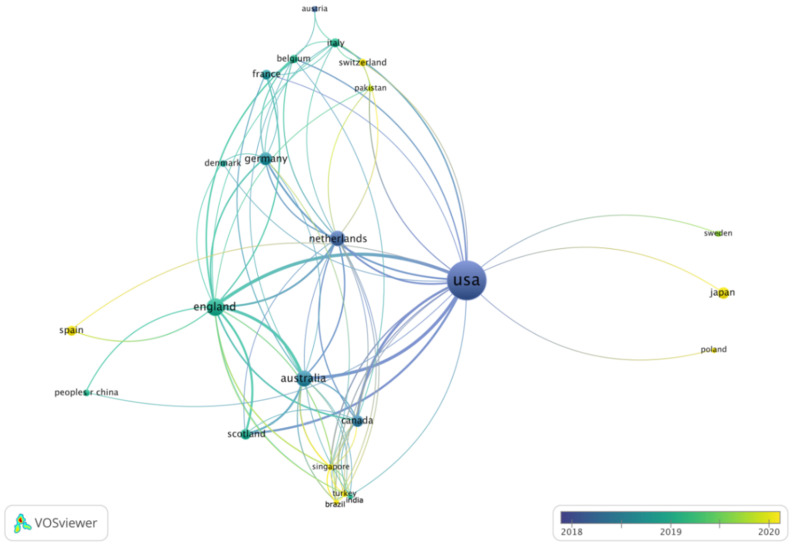
Collaborations between countries among articles on post-intensive care syndrome. Co-authorship-based network. Lines between countries indicate direct links (i.e., co-authorships). Thicker lines are indicative of stronger links (i.e., more co-authorships). The further two countries are apart, the weaker is their relation. Colors indicate the average publication year of a country’s articles. A number of 26 countries with at least three publications were identified. Greece, Portugal, and South Korea were excluded as they did not have any connection to the network. Created using VOSviewer.

We identified 26 countries with at least three publications (Figure 3). As Greece, Portugal, and South Korea did not bear connections to the network, they were not displayed. The US lies in the network’s center and has collaborations with 21 countries, followed by the Netherlands (14 links), Australia (12 links), England (17 links), Canada (10 links), and Germany (nine links). With an average publication year of 2018, articles from the US and the Netherlands were published earlier than articles from other countries.

Each of the 20 countries with the highest number of PICS articles has increased its research output since 2012 (Table 2). With 203 publications, most articles on PICS were published by authors affiliated with US institutions, which is also reflected by the relative US research activity during the three time periods of 3.9, 10.7, and 13.4, respectively. At the same time, the median CNCI of US-affiliated articles decreased from 0.8 (IQR 0.3; 2.6) in 2012–2014 to 0.4 (IQR 0.0; 1.0) in 2018–2021. Similar to the US, the relative research activity of England, Australia, the Netherlands, and Germany increased in 2018–2021 compared to previous years. Authors affiliated with institutions from various other countries entered the research field after 2015, for example, authors affiliated with institutions from Spain, Japan, and Italy.

### 3.4. Bibliometric Analysis by Institution

**Figure 4:**
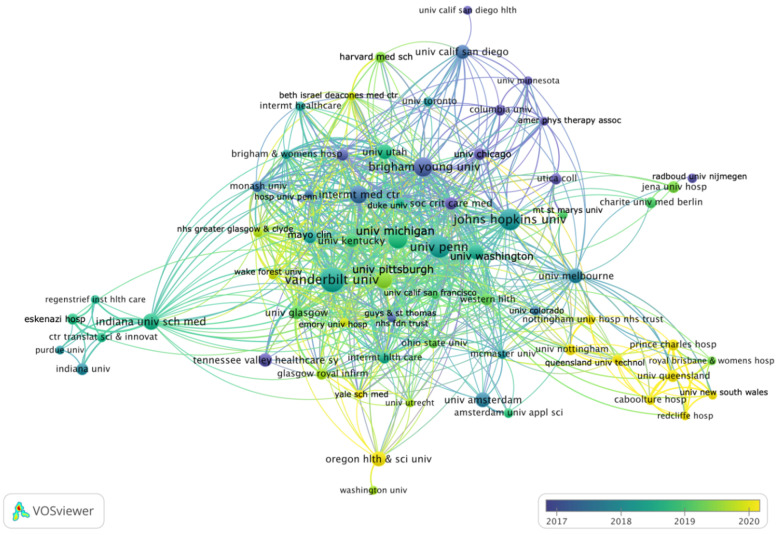
Collaborations between institutions among articles on post-intensive care syndrome. Co-authorship-based network. Lines between institutions indicate direct links (i.e., co-authorships). Thicker lines are indicative of stronger links (i.e., more co-authorships). The further two institutions are apart, the weaker is their relation. Colors indicate the average publication year of an institution’s articles. Institutions with at least four publications are shown. Five institutions were excluded as they did not show any connection with the network. Created using VOSviewer.

As indicated in the highly linked network, we found a large number of research collaborations between institutions (Figure 4). At the center of the network, Johns Hopkins University and the University of Pennsylvania have the most institutional links (44 each), followed by Brigham Young University (43 links). VOSviewer identified nine clusters, most of which are formed around US-based institutions. The exceptions are clusters around the University of Nottingham (England) and the University of Queensland (Australia), as well as other institutions, for example, Charité—Universitätsmedizin Berlin (Germany), the University of Amsterdam (the Netherlands), the University of Melbourne (Australia), McMaster University (Canada), or the University of Glasgow (Scotland).

Apart from the main cluster, institutions from the US state of Indiana have formed a separate collaborative network, primarily linked to the main cluster via collaborations of the Indiana University School of Medicine. When we analyzed collaborations between individual authors, we also observed a separate, Indiana-based cluster (Figure S1).

Recent collaborations (yellow circles in the periphery of the network in Figure 4) have formed around the Oregon Health and Science University and Yale School of Medicine in the US, as well as the University of Nottingham, Nottingham University Hospitals NHS Trust, the University of Queensland, Caboolture Hospital, Prince Charles Hospital, the University of New South Wales, Queensland University of Technology, and Redcliffe Hospital in Australia and the United Kingdom. A separate, recently formed collaborative network of authors from Oregon (TA Hall and K Bradbury, among others) also appeared in the collaborative author network (Figure S1).

### 3.5. Keyword Co-Occurrence Network

**Figure 5:**
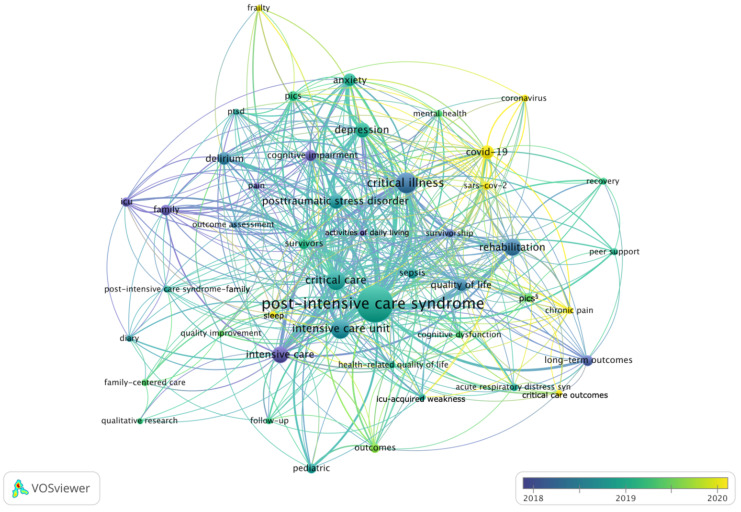
Keyword co-occurrence among articles on post-intensive care syndrome. Lines indicate direct keyword co-occurrences. Thicker lines are indicative for more direct keyword co-occurrences. The further two keywords are apart, the weaker is their relation. Colors indicate the average publication year of articles with the respective keyword. Keywords with at least five publications are shown. ^§^ Abbreviation for the keyword ‘post-intensive care syndrome (pics)’. Created using VOSviewer.

The keyword ‘post-intensive care syndrome’ lies in the center of the keyword co-occurrence network (Figure 5). Keywords with most co-occurrences apart from PICS are ‘critical care’, ‘intensive care unit’, ‘intensive care’, and ‘critical illness’. The visual overlay of the average publication year of keywords allows for the identification of trends over time. Early keywords with an average publication year of 2018 include ‘family’, ‘pain’, ‘cognitive impairment’, and ‘activities of daily living’. Keywords on mental health impairments such as ‘anxiety’, ‘depression’, and ‘posttraumatic stress disorder’, as well as ‘ICU-acquired weakness’ and ‘health-related quality of life’ center around 2019. Starting with the emergence of the COVID-19 pandemic in 2020, COVID-19-related keywords have entered the PICS research field. More recently, the keywords ‘frailty’, ‘sleep’, and ‘chronic pain’ have been used in the context of PICS, indicating new aspects of research in the field.

### 3.6. Characteristics of the 100 Most-Cited Articles on Post-Intensive Care Syndrome

Half of the 100 most-cited articles on PICS were original research articles (50 articles), 42 articles were reviews, seven articles were editorials/letters, and one article was a study protocol (see Table S1 for full list). More than half of the articles originate from the US, followed by the Netherlands (eight articles), Australia (seven articles), and England (six articles) (Table 3). With 21 articles, *Critical Care Medicine* was the most popular journal, followed by *AACN Advanced Critical Care* (eight articles), *Critical Care*, and *Current Opinion in Critical Care* (both five articles) (Table 4).

The most-cited article on PICS is the report from the Society of Critical Care Medicine conference that initially introduced the PICS terminology [11] (Table 5). The report from the second stakeholder meeting of the Society of Critical Care Medicine on this topic received the second most citations among original research articles [13]. The other top ten original research articles were published in 2016 or later [22,23,24,25,26,27,28,29], with two recent articles pertaining to COVID-19 [22,25] and one article pertaining to the establishment of an ICU recovery center [27]. The most frequently cited review on PICS discusses the ramifications of critical illness for family members (Table 6) [30]. Another top ten review also discusses repercussions for the families of ICU patients [31]. Two reviews are dedicated to measures for PICS prevention and treatment (ICU bundles and rehabilitation) [32,33], four reviews cover PICS in general [34,35,36,37], one review discusses PICS in pediatric ICU patients [38], and one recent review is on COVID-19 [39].

## 4. Discussion

Since the introduction of the PICS terminology, research output in the field has increased exponentially from 11 articles in 2012 to 95 articles in 2020. While the umbrella term PICS was introduced in 2012 [11], researchers in critical care had already demanded for research on the frequent functional impairments after critical illness in the early 2000s [10]. The stark increase in PICS research output in recent years, particularly after 2017, reveals that the research community has indeed acted upon these demands, albeit with a delay of more than ten years.

Most publications originate from the US—203 of 373 articles in our sample were written by authors affiliated with US institutions—followed by England, Australia, the Netherlands, and Germany (Table 7). The relative research output of these countries has increased from 2012–2014 to 2018–2021, as indicated by above-average relative research activities. These five countries are also in the center of the co-authorship-based collaboration network. On an institutional level, we identified a separate cluster around Indiana-based institutions, and a recently formed cluster of collaboration among institutions from Australia and England. The predominant role of a few high-income countries implies that the knowledge on PICS and the trajectories of post-ICU care stem primarily from highly developed health care systems in the US and Europe. As there are large global discrepancies in the organization of critical care, available resources, quality of acute as well as post-ICU care, and patient characteristics [40], our analysis uncovers the need for more diverse PICS research outside of Europe and the US. Studies in Asia, Africa, and South America could help validate existing findings on the epidemiology of PICS, risk factors, and effective treatment options. In this context, it is a positive development that authors affiliated with institutions from a more diverse set of countries, such as China, Pakistan, and Turkey have entered the PICS research stage in recent years. The newcomers usually collaborate with established institutions and authors from the US and Europe.

Our analysis of keyword co-occurrence unveils past and recent trends in PICS research. Not surprisingly, we reveal that common keywords pertain to cognition, mental health, physical health, and quality of life. As an interesting finding, articles with keywords on cognitive impairment center on 2018, whereas the keywords on mental health impairment (e.g., depression or anxiety) and physical impairment center on 2019. Notably, family was already a common keyword in early PICS publications, and the second most-cited PICS publication from 2012 pertains to PICS-F [30]. Very recently, COVID-19-related keywords have entered the PICS research field. Two of the ten most-cited original research articles [22,25] and one of the ten most-cited reviews [39] is about COVID-19, which underlines the highly dynamic and rapidly evolving research at the intersection of COVID-19 and PICS.

The most-cited article in the field is the stakeholder conference report by Needham et al. [11], which initially introduced the PICS terminology. It was published in *Critical Care Medicine*, which is the most common outlet for highly-cited PICS research, with 21 of the 100 most-cited articles published in this journal.

While many keywords and most-cited publications explored epidemiological aspects of PICS, we found very few keywords and articles on effective ways to prevent or counteract PICS. The only keywords in our network on PICS prevention and treatment were ‘diary’, ‘family-centered care’, and ‘peer support’. One top-cited review focused on ICU bundle implementation to prevent PICS [33], one top-cited review focused on early rehabilitation [32], and one top-cited original research article illustrated the establishment of an ICU recovery center [27]. Our analysis demonstrates the pressing need for sound evidence on effective measures for PICS prevention, PICS treatment, and organization of post-ICU care—a demand that is mirrored by recent reviews [41,42].

Several limitations of this study warrant consideration. Most importantly, only the Web of Science, which allowed for the extensive export of bibliometric data, was searched for articles on PICS. Not all research articles are indexed in the Web of Science. Thus, articles and citations that were indexed in other databases were not included in this study. The Web of Science, however, was searched using multiple spelling variants of PICS. Second, research on long-term impairments in ICU survivors had already been conducted before the introduction of the PICS terminology [10], for example, in a Dutch ICU study from 1988 with a two-year follow-up [9]. Hence, some articles on post-ICU impairments that were published after 2012 might not have used the PICS terminology and could have been missed by our search strategy. By the same token, the observed increase in PICS research output might possibly be due to an incremental establishment of the term PICS in an already existent research field. However, various consensus conferences have reiterated the lack of research on long-term functional impairments in ICU patients [4,11,13]. Third, we used the CNCI to determine the normalized citations of individual publications. The CNCI puts an article’s citations in relation to the average number of citations of articles of the same document type, year, and research field [19]. As the CNCI is, by definition, influenced by outliers (e.g., few highly-cited articles), it should be interpreted with caution in the case of small sample sizes (such as the period 2012–2015). Finally, articles that have been published longer have had more time to accumulate citations [43]. To account for this bias, we calculated the CNCI, which is independent of an article’s age, and report articles’ citations in the year of publication and the two following years. Furthermore, two of the ten most-cited original articles and two of the ten most-cited reviews were published in 2019 or later, which might indicate that time bias could be less relevant in the relatively young PICS research field.

## 5. Conclusions

Our bibliometric network analysis of Web of Science-indexed PICS publications shows a sharp increase in publication output since 2017. Most articles originate from US-based institutions and authors, followed by England, Australia, the Netherlands, and Germany. We found strong collaborations between different countries, institutions, and individuals, with a recent formation of a collaborative network of English and Australian institutions. Article keywords pertain to various aspects of PICS domains, and more recently, COVID-19. Only a few keywords and highly-cited articles, however, explore interventions to prevent or treat PICS. Our analysis maps out a highly dynamic and growing research field, predominantly with contributors from the US and Europe.

## Figures and Tables

**Table 1:** Articles on post-intensive care syndrome, by article type.

Article Type	Articles, *n*
Original research	145
Review	103
Editorial or letter	58
Meeting abstract	33
Protocol paper	19
Case report	9
Research letter	5
Book review	1

**Table 2:** Research output, Category Normalized Citation Index, and relative research activity, by country and time period.

Country	2012–2014			2015–2017			2018–2021			All Years
	N	CNCI ^§^	RRA	N	CNCI ^§^	RRA	N	CNCI ^§^	RRA	N
USA	14	0.8 (0.3; 2.6)	3.9	56	0.5 (0.1; 1.4)	10.7	133	0.4 (0.0; 1.0)	13.4	203
England	2	2.4 (1.0; 3.9)	0.6	5	0.7 (0.7; 2.1)	1.0	35	0.5 (0.2; 2.3)	3.5	42
Australia	3	1.0 (0.3; 1.0)	0.8	7	1.1 (0.3; 2.3)	1.3	24	0.9 (0.0; 2.2)	2.4	34
Netherlands	4	0.4 (0.0; 0.9)	1.1	5	1.3 (0.7; 1.4)	1.0	23	0.7 (0.1; 1.6)	2.3	32
Germany	3	0.4 (0.0; 1.0)	0.8	2	0.8 (0.1; 1.4)	0.4	18	0.3 (0.0; 0.8)	1.8	23
Japan	0			1	0.3 (0.3; 0.3)	0.2	17	0.2 (0.0; 0.8)	1.7	18
Canada	0			4	2.1 (1.7; 2.3)	0.8	12	1.2 (0.3; 2.1)	1.2	16
Scotland	0			2	1.9 (0.4; 3.4)	0.4	12	2.2 (0.7; 3.6)	1.2	14
France	1	0.9 (0.9; 0.9)	0.3	2	1.2 (1.1; 1.3)	0.4	10	0.3 (0.0; 2.4)	1.0	13
Spain	0			0			12	0.0 (0.0; 0.1)	1.2	12
Italy	1	0.1 (0.1; 0.1)	0.3	0			9	0.0 (0.0; 2.9)	0.9	10
Belgium	0			2	1.7 (1.3; 2.1)	0.4	7	0.7 (0.0; 2.4)	0.7	9
South Korea	0			1	1.1 (1.1; 1.1)	0.2	7	0.4 (0.0; 0.8)	0.7	8
Switzerland	0			0			7	0.5 (0.0; 2.9)	0.7	7
China	0			1	0.4 (0.4; 0.4)	0.2	5	0.2 (0.2; 0.4)	0.5	6
Denmark	0			1	0.9 (0.9; 0.9)	0.2	5	0.3 (0.0; 0.3)	0.5	6
Pakistan	0			0			5	0.3 (0.2; 0.5)	0.5	5
Singapore	0			0			5	0.3 (0.0; 0.5)	0.5	5
Turkey	0			0			5	0.2 (0.0; 0.9)	0.5	5
Sweden	0			1	0.3 (0.3; 0.3)	0.2	4	0.2 (0.0; 2.0)	0.4	5

Countries with at least five publications shown. **^§^** Median (IQR). Depending on authors’ affiliations, one article may be assigned to multiple countries. CNCI, Category Normalized Citation Index; RRA, relative research activity.

**Table 3:** Countries of origin of the 100 most-cited articles on post-intensive care syndrome.

Country	Articles, *n*
USA	52
Netherlands	8
Australia	7
England	6
Scotland	5
France	4
Germany	4
Japan	3
Canada	2
South Korea	2
Denmark	2

Countries with at least two top-100 publications shown. The corresponding author’s institutional affiliation defined the country of origin.

**Table 4:** Publishing journals of the 100 most-cited articles on post-intensive care syndrome.

Rank	Journal	Impact Factor 2020 *	Articles, *n*
1	Critical Care Medicine	7.598	21
2	AACN Advanced Critical Care	^- §^	8
3	Critical Care	9.097	5
	Current Opinion in Critical Care	3.687	5
4	Journal of Critical Care	3.425	4
	Annals of the American Thoracic Society	6.831	4
5	Intensive Care Medicine	17.440	3
	Physical Therapy	3.140 ^†^	3
	Seminars in Respiratory and Critical Care Medicine	3.119	3
6	Annals of Intensive Care	6.925	2
	BMJ Open	2.692	2
	British Journal of Anaesthesia	9.166	2
	Journal of Rehabilitation Medicine	2.912	2
	Rehabilitation Psychology	2.564	2
	Pediatric Critical Care Medicine	3.624	2

Journals with at least two top-100 publications shown. * Based on Clarivate Analytics Journal Citation Reports 2020 [21]. ^§^ *AACN Advanced Critical Care* has not received an impact factor yet. ^†^ The latest impact factor of *Physical Therapy* is from 2019.

**Table 5:** Most-cited original research articles on post-intensive care syndrome, ordered by citations.

Rank	Year	Title of Original Research Article	First Author	Citations	Citations (First Three Years)
1	2012	Improving long-term outcomes after discharge from intensive care unit: Report from a stakeholders’ conference	DM Needham	939	91
2	2014	Exploring the scope of post-intensive care syndrome therapy and care: Engagement of non-critical care providers and survivors in a second stakeholders meeting	D Elliott	206	42
3	2021	Postdischarge symptoms and rehabilitation needs in survivors of COVID-19 infection: A cross-sectional evaluation	SJ Halpin	162	-
4	2018	Co-occurrence of post-intensive care syndrome problems among 406 survivors of critical illness	A Marra	86	50
5	2018	Anxiety, depression and post traumatic stress disorder after critical illness: A UK-wide prospective cohort study	R Hatch	83	35
6	2020	Rehabilitation and respiratory management in the acute and early post-acute phase: Instant paper from the field on rehabilitation answers to the COVID-19 emergency	C Kiekens	50	-
7	2018	Determinants of long-term outcome in ICU survivors: Results from the FROG-ICU study	E Gayat	49	33
8	2018	Comprehensive care of ICU survivors: Development and implementation of an ICU recovery center	CM Sevin	47	35
9	2016	Resilience in survivors of critical illness in the context of the survivors’ experience and recovery	JH Maley	46	13
10	2016	Surviving critical illness: What is next? An expert consensus statement on physical rehabilitation after hospital discharge	ME Major	44	14

**Table 6:** Most-cited reviews on post-intensive care syndrome, ordered by citations.

Rank	Year	Title of Review	First Author	Citations	Citations (First Three Years)
1	2012	Family response to critical illness: Postintensive care syndrome-family	JE Davidson	396	40
2	2020	COVID-19: ICU delirium management during SARS-CoV-2 pandemic	K Kotfis	163	-
3	2017	The ABCDEF bundle: Science and philosophy of how ICU liberation serves patients and families	W Ely	152	69
4	2017	Post-intensive care syndrome: An overview	G Rawal	135	34
5	2014	Rehabilitation interventions for postintensive care syndrome: A systematic review	J Mehlhorn	125	35
6	2012	Having a loved one in the ICU: The forgotten family	M Schmidt	87	10
7	2018	Conceptualizing post intensive care syndrome in children—The PICS-p framework	JC Manning	74	48
8	2019	Post-intensive care syndrome: Its pathophysiology, prevention, and future directions	S Inoue	72	-
9	2016	Postintensive care syndrome: Right care, right now … and later	MA Harvey	71	19
10	2012	The burdens of survivorship: An approach to thinking about long-term outcomes after critical illness	TJ Iwashyna	69	12

**Table 7:** Aspects of the current PICS research field.

Strengths	Limitations
- We observe a stark increase in PICS research output in recent years.	Few of the highly-cited publications pertain to measures of PICS treatment or prevention.
- Since 2015, publications from a broader array of countries have been entering the research field, e.g., from Spain, Italy, China, or Japan.	Most publications originate from few high-developed countries, namely the US, England, Australia, the Netherlands, and Germany.
- Network analysis shows a high level of collaboration among institutions and individual authors.	
- Research has focused on all three PICS domains, namely impairments of cognitive functions, mental health, and physical functions.	
- Responding to the COVID-19 pandemic, studies at the intersection of COVID-19 and PICS have been emerging.	

PICS, post-intensive care syndrome.

## Data Availability

The data presented in this study are available on request from the corresponding author.

## Supplementary Materials

The following supporting information can be downloaded at: https://www.mdpi.com/article/10.3390/medicina58020170/s1, Figure S1: Collaboration between authors of articles on post-intensive care syndrome, Table S1: One hundred most-cited articles on post-intensive care syndrome, ordered by number of total citations.

## References

[1] Halpern N.A., Pastores S.M. (2010). Critical care medicine in the United States 2000–2005: An analysis of bed numbers, occupancy rates, payer mix, and costs. Crit. Care Med..

[2] Lilly C.M., Swami S., Liu X., Riker R.R., Badawi O. (2017). Five-year trends of critical care practice and outcomes. Chest.

[3] Kaukonen K.-M., Bailey M., Suzuki S., Pilcher D., Bellomo R. (2014). Mortality related to severe sepsis and septic shock among critically ill patients in Australia and New Zealand, 2000–2012. JAMA.

[4] Angus D.C., Carlet J. (2003). Surviving intensive care: A report from the 2002 Brussels Roundtable. Intensive Care Med..

[5] Crawford S.W., Petersen F.B. (1992). Long-term survival from respiratory failure after marrow transplantation for malignancy. Am. Rev. Respir. Dis..

[6] Perl T.M., Dvorak L., Hwang T., Wenzel R.P. (1995). Long-term survival and function after suspected gram-negative sepsis. JAMA.

[7] Franklin C., Jackson D. (1983). Discharge decision-making in a medical ICU: Characteristics of unexpected readmissions. Crit. Care Med..

[8] Bürgisser C., Ritz R. (1982). Follow-up of intensive medical care patients. Schweiz. Med. Wochenschr..

[9] Jacobs C.J., van der Vliet J.A., van Roozendaal M.T., van der Linden C.J. (1988). Mortality and quality of life after intensive care for critical illness. Intensive Care Med..

[10] Angus D.C., Carlet J. (2003). Surviving Intensive Care.

[11] Needham D.M., Davidson J., Cohen H., Hopkins R.O., Weinert C., Wunsch H., Zawistowski C., Bemis-Dougherty A., Berney S.C., Bienvenu O.J. (2012). Improving long-term outcomes after discharge from intensive care unit: Report from a stakeholders’ conference. Crit. Care Med..

[12] Nolan J.P., Neumar R.W., Adrie C., Aibiki M., Berg R.A., Böttiger B.W., Callaway C., Clark R.S.B., Geocadin R.G., Jauch E.C. (2008). Post-cardiac arrest syndrome: Epidemiology, pathophysiology, treatment, and prognostication: A scientific statement from the International Liaison Committee on Resuscitation; The American Heart Association Emergency Cardiovascular Care Committee; The Council on Cardiovascular Surgery and Anesthesia; The Council on Cardiopulmonary, Perioperative, and Critical Care; The Council on Clinical Cardiology; The Council on Stroke. Resuscitation.

[13] Elliott D., Davidson J.E., Harvey M.A., Bemis-Dougherty A., Hopkins R.O., Iwashyna T.J., Wagner J., Weinert C., Wunsch H., Bienvenu O.J. (2014). Exploring the scope of post–intensive care syndrome therapy and care: Engagement of non-critical care providers and survivors in a second stakeholders meeting. Crit. Care Med..

[14] Zare-Farashbandi F., Geraei E., Siamaki S. (2014). Study of co-authorship network of papers in the Journal of Research in Medical Sciences using social network analysis. J. Res. Med. Sci..

[15] Newman M.E.J. (2001). The structure of scientific collaboration networks. Proc. Natl. Acad. Sci. USA.

[16] Van Eck N.J., Waltman L. (2013). VOSviewer Manual.

[17] Van Eck N.J., Waltman L., Decker R., Lenz H.J. (2007). VOS: A new method for visualizing similarities between objects. Advances in Data Analysis.

[18] Shvindina H. (2019). Coopetition as an emerging trend in research: Perspectives for safety & security. Safety.

[19] Szomszor M., Adams J., Fry R., Gebert C., Pendlebury D.A., Potter R.W.K., Rogers G. (2021). Interpreting bibliometric data. Front. Res. Metr. Anal..

[20] Xie L., Lu B., Ma Y., Yin J., Zhai X., Chen C., Xie W., Zhang Y., Zheng L., Li P. (2021). The 100 most-cited articles about the role of neurovascular unit in stroke 2001–2020: A bibliometric analysis. CNS Neurosci. Ther..

[21] Clarivate Journal Citation Reports. https://jcr.clarivate.com/jcr/home.

[22] Halpin S.J., McIvor C., Whyatt G., Adams A., Harvey O., McLean L., Walshaw C., Kemp S., Corrado J., Singh R. (2021). Postdischarge symptoms and rehabilitation needs in survivors of COVID-19 infection: A cross-sectional evaluation. J. Med. Virol..

[23] Marra A., Pandharipande P.P., Girard T.D., Patel M.B., Hughes C.G., Jackson J.C., Thompson J.L., Chandrasekhar R., Ely E.W., Brummel N.E. (2018). Co-occurrence of post-intensive care syndrome problems among 406 survivors of critical illness. Crit. Care Med..

[24] Hatch R., Young D., Barber V., Griffiths J., Harrison D.A., Watkinson P. (2018). Anxiety, depression and post traumatic stress disorder after critical illness: A UK-wide prospective cohort study. Crit. Care.

[25] Kiekens C., Boldrini P., Andreoli A., Avesani R., Gamna F., Grandi M., Lombardi F., Lusuardi M., Molteni F., Perboni A. (2020). Rehabilitation and respiratory management in the acute and early post-acute phase. “Instant paper from the field” on rehabilitation answers to the COVID-19 emergency. Eur. J. Phys. Rehabil. Med..

[26] Gayat E., Cariou A., Deye N., Vieillard-Baron A., Jaber S., Damoisel C., Lu Q., Monnet X., Rennuit I., Azoulay E. (2018). Determinants of long-term outcome in ICU survivors: Results from the FROG-ICU study. Crit. Care.

[27] Sevin C.M., Bloom S.L., Jackson J.C., Wang L., Ely E.W., Stollings J.L. (2018). Comprehensive care of ICU survivors: Development and implementation of an ICU recovery center. J. Crit. Care.

[28] Maley J.H., Brewster I., Mayoral I., Siruckova R., Adams S., McGraw K.A., Piech A.A., Detsky M., Mikkelsen M.E. (2016). Resilience in survivors of critical illness in the context of the survivors’ experience and recovery. Ann. Am. Thorac. Soc..

[29] Major M.E., Kwakman R., Kho M.E., Connolly B., McWilliams D., Denehy L., Hanekom S., Patman S., Gosselink R., Jones C. (2016). Surviving critical illness: What is next? An expert consensus statement on physical rehabilitation after hospital discharge. Crit. Care.

[30] Davidson J.E., Jones C., Bienvenu O.J. (2012). Family response to critical illness: Postintensive care syndrome-family. Crit. Care Med..

[31] Schmidt M., Azoulay E. (2012). Having a loved one in the ICU: The forgotten family. Curr. Opin. Crit. Care.

[32] Mehlhorn J., Freytag A., Schmidt K., Brunkhorst F.M., Graf J., Troitzsch U., Schlattmann P., Wensing M., Gensichen J. (2014). Rehabilitation interventions for postintensive care syndrome: A systematic review. Crit. Care Med..

[33] Ely E.W. (2017). The ABCDEF bundle: Science and philosophy of how ICU liberation serves patients and families. Crit. Care Med..

[34] Iwashyna T.J., Netzer G. (2012). The burdens of survivorship: An approach to thinking about long-term outcomes after critical illness. Semin. Respir. Crit. Care Med..

[35] Rawal G., Yadav S., Kumar R. (2017). Post-intensive care syndrome: An overview. J. Transl. Int. Med..

[36] Inoue S., Hatakeyama J., Kondo Y., Hifumi T., Sakuramoto H., Kawasaki T., Taito S., Nakamura K., Unoki T., Kawai Y. (2019). Post-intensive care syndrome: Its pathophysiology, prevention, and future directions. Acute Med. Surg..

[37] Harvey M.A., Davidson J.E. (2016). Postintensive care syndrome: Right care, right now…and later. Crit. Care Med..

[38] Manning J.C., Pinto N.P., Rennick J.E., Colville G., Curley M.A.Q. (2018). Conceptualizing post intensive care syndrome in children—The PICS-p framework. Pediatr. Crit. Care Med..

[39] Kotfis K., Williams Roberson S., Wilson J.E., Dabrowski W., Pun B.T., Ely E.W. (2020). COVID-19: ICU delirium management during SARS-CoV-2 pandemic. Crit. Care.

[40] Adhikari N.K., Fowler R.A., Bhagwanjee S., Rubenfeld G.D. (2010). Critical care and the global burden of critical illness in adults. Lancet.

[41] Azoulay E., Vincent J.-L., Angus D.C., Arabi Y.M., Brochard L., Brett S.J., Citerio G., Cook D.J., Curtis J.R., Dos Santos C.C. (2017). Recovery after critical illness: Putting the puzzle together—A consensus of 29. Crit. Care.

[42] Rousseau A.-F., Prescott H.C., Brett S.J., Weiss B., Azoulay E., Creteur J., Latronico N., Hough C.L., Weber-Carstens S., Vincent J.-L. (2021). Long-term outcomes after critical illness: Recent insights. Crit. Care.

[43] Pepe A., Kurtz M.J. (2012). A measure of total research impact independent of time and discipline. PLoS ONE.

