# Supplementary material for: A Decade of Post-Intensive Care Syndrome: A Bibliometric Network Analysis
Source: Medicina (Kaunas). 2022 Jan 23;58(2):170. doi: 10.3390/medicina58020170 (PMC8880008; doi:10.3390/medicina58020170)
Supplement: Supplementary file 1 [file medicina-58-00170-s001.zip › medicina-1516477-supplementary.pdf]

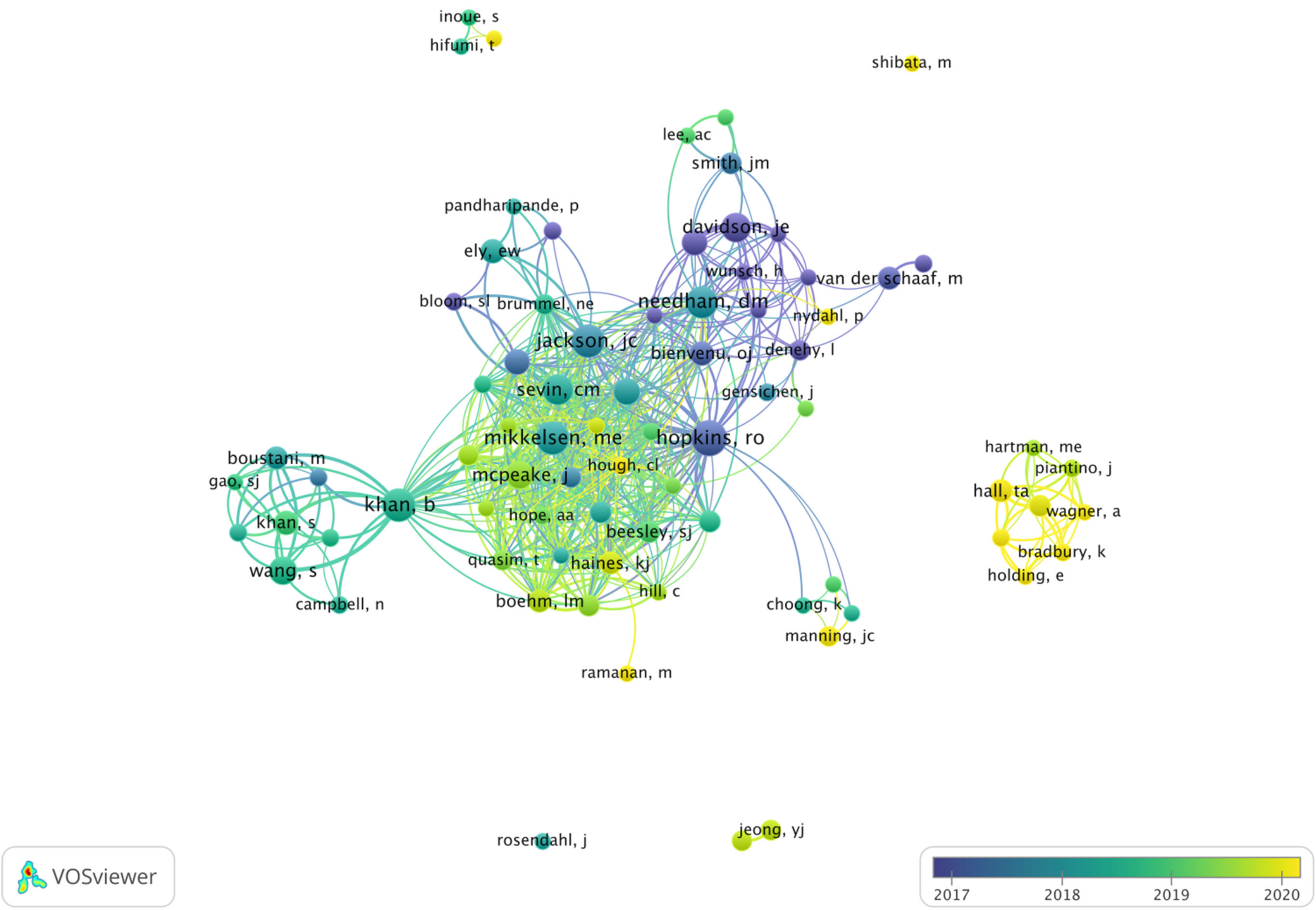

Figure S1. Collaborations between authors of articles on post-intensive care syndrome. Co-authorship-based network. Lines between authors indicate direct links (i.e., co-authorships). Thicker lines are indicative for stronger links (i.e., more co-authorships). The further authors are apart, the weaker is their relation. Colors indicate the average year of the publications of the respective author. A number of 78 authors with at least four publications appear in the network, of which 15 do not have a connection to the main network. Created using VOSviewer.

Table S1. One hundred most-cited articles on post-intensive care syndrome, ordered by the number of total citations.

Rank	Year	Title	First Author	Article Type	Total Citations	Citations (First Three Years)
1	2012	Improving long-term outcomes after discharge from intensive care unit: Report from a stakeholders' conference	DM Needham	Original	939	91
2	2012	Family response to critical illness: Postintensive care syndrome-family	JE Davidson	Review	396	40
3	2014	Exploring the scope of post-intensive care syndrome therapy and care: Engagement of non-critical care providers and survivors in a second stakeholders meeting	D Elliott	Original	206	42
4	2020	COVID-19: ICU delirium management during SARS-CoV-2 pandemic	K Kotfis	Review	163	165
5	2021	Postdischarge symptoms and rehabilitation needs in survivors of COVID-19 infection: A cross-sectional evaluation	SJ Halpin	Original	162	150
6	2017	The ABCDEF bundle: Science and philosophy of how ICU liberation serves patients and families	EW Ely	Review	152	69
7	2017	Post-intensive care syndrome: An overview	G Rawal	Review	135	34
8	2014	Rehabilitation interventions for postintensive care syndrome: A systematic review	J Mehlhorn	Review	125	35
9	2012	Having a loved one in the ICU: The forgotten family	M Schmidt	Review	87	10
10	2018	Co-occurrence of post-intensive care syndrome problems among 406 survivors of critical illness*	A Marra	Original	86	50
11	2018	Anxiety, depression and post traumatic stress disorder after critical illness: A UK-wide prospective cohort study	R Hatch	Original	83	35
12	2020	Covid-19 and post intensive care syndrome: A call for action	HJ Stam	Editorial	80	77
13	2018	Conceptualizing post intensive care syndrome in children - The PICS-p framework	JC Manning	Review	74	48
14	2019	Post-intensive care syndrome: Its pathophysiology, prevention, and future directions	S Inoue	Review	72	67
15	2016	Postintensive care syndrome: Right care, right now ... and later	MA Harvey	Review	71	19

16	2012	The burdens of survivorship: An approach to thinking about long-term outcomes after critical illness	TJ Iwashyna	Review	69	12
17	2013	Implementation of the pain, agitation, and delirium clinical practice guidelines and promoting patient mobility to prevent post-intensive care syndrome	JE Davidson	Review	53	7
18	2018	Life after critical illness in children - Toward an understanding of pediatric post-intensive care syndrome	RS Watson	Review	51	35
19	2020	Rehabilitation and respiratory management in the acute and early post-acute phase. “Instant paper from the field” on rehabilitation answers to the COVID-19 emergency	C Kiekens	Original	50	46
20	2018	Determinants of long-term outcome in ICU survivors: Results from the FROG-ICU study	E Gayat	Original	49	33
21	2018	Early rehabilitation to prevent postintensive care syndrome in patients with critical illness: A systematic review and meta-analysis	R Fuke	Review	48	29
22	2018	Comprehensive care of ICU survivors: Development and implementation of an ICU recovery center	CM Sevin	Original	47	35
23	2016	Peer support as a novel strategy to mitigate post-intensive care syndrome	ME Mikkelsen	Review	47	22
24	2016	Resilience in survivors of critical illness in the context of the survivors' experience and recovery	JH Maley	Original	46	13
25	2019	The effect of ICU diaries on psychological outcomes and quality of life of survivors of critical illness and their relatives: A systematic review and meta-analysis	PA McIlroy	Review	46	43
26	2016	Surviving critical illness: What is next? An expert consensus statement on physical rehabilitation after hospital discharge	ME Major	Original	44	14
27	2015	CE: Critical care recovery center: An innovative collaborative care model for ICU survivors	BA Khan	Original	44	11
28	2015	Transforming PICU culture to facilitate early rehabilitation	RO Hopkins	Review	40	6
29	2018	Physical impairments associated with post-intensive care syndrome: Systematic Review based on the World Health Organization's International Classification of Functioning, Disability and Health framework	PJ Ohtake	Review	38	19

30	2019	Approaches to addressing post-intensive care syndrome among intensive care unit survivors. A narrative review	SM Brown	Review	36	36
31	2018	Follow-up services for improving long-term outcomes in intensive care unit (ICU) survivors	OJ Schofield-Robinson	Review	35	17
32	2018	Perspectives of survivors, families and researchers on key outcomes for research in acute respiratory failure	VD Dinglas	Original	34	26
33	2015	Recommendations for intensive care follow-up clinics; report from a survey and conference of Dutch intensive cares	M Van der Schaaf	Original	34	13
34	2017	Post-ICU symptoms, consequences, and follow-up: An integrative review	H Svenningsen	Review	33	18
35	2019	Models of peer support to remediate post-intensive care syndrome: A report developed by the Society of Critical Care Medicine Thrive International Peer Support Collaborative	J McPeake	Original	32	32
36	2015	Physical, cognitive, and psychological disability following critical illness: What is the risk?	JE Jutte	Review	31	9
37	2018	Preventing posttraumatic stress in icu survivors: A single-center pilot randomized controlled trial of ICU diaries and psychoeducation*	MS Kredentser	Original	31	20
38	2019	A computer vision system for deep learning-based detection of patient mobilization activities in the ICU	S Yeung	Original	29	29
39	2016	Predictors of posttraumatic stress and quality of life in family members of chronically critically ill patients after intensive care	GB Wintermann	Original	29	8
40	2016	A clinic model: Post-intensive care syndrome and post-intensive care syndrome-family	EL Huggins	Original	28	16
41	2017	Muscle mass and physical recovery in ICU: Innovations for targeting of nutrition and exercise	PE Wischmeyer	Review	28	15
42	2016	Caregiver strain and posttraumatic stress symptoms of informal caregivers of intensive care unit survivors	SA van den Born-van Zanten	Original	27	9
43	2018	Long-term outcome after the acute respiratory distress syndrome: Different from general critical illness?	T Bein	Review	27	14

44	2012	Medical and economic implications of cognitive and psychiatric disability of survivorship	RO Hopkins	Review	26	6
45	2012	Strategies for post ICU rehabilitation	L Denehy	Review	26	6
46	2012	The truth about consequences - Post-intensive care syndrome in intensive care unit survivors and their families	MA Harvey	Editorial	26	3
47	2019	Enablers and barriers to implementing ICU follow-up clinics and peer support groups following critical illness: The Thrive Collaboratives	KJ Haines	Original	25	25
48	2020	Home and community-based physical therapist management of adults with post-intensive care syndrome	JM Smith	Review	25	24
49	2016	Post-ICU syndrome: Rescuing the undiagnosed	EA Myers	Review	25	4
50	2016	Implementing a mobility program to minimize post-intensive care syndrome	RO Hopkins	Review	24	6
51	2016	Patient and family post-intensive care syndrome	JE Davidson	Editorial	24	5
52	2019	Post-intensive care syndrome: Impact, prevention, and management	GA Colbenson	Editorial	23	23
53	2016	Reappraisal of visiting policies and procedures of patient's family information in 188 French ICUs: A report of the Outcomerea Research Group	M Garrouste-Orgeas	Original	23	10
54	2020	Chronic pain after COVID-19: Implications for rehabilitation	HI Kemp	Editorial	22	21
55	2018	Critical care pharmacists and medication management in an ICU recovery center	JL Stollings	Original	22	21
56	2020	Society of Critical Care Medicine's International Consensus Conference on prediction and identification of long-term impairments after critical illness	ME Mikkelsen	Original	21	22
57	2019	Key mechanisms by which post-ICU activities can improve in-ICU care: Results of the international THRIVE collaboratives	KJ Haines	Original	20	20
58	2018	Improving health care for critically ill patients using an evidence-based collaborative approach to ABCDEF bundle dissemination and implementation	MA Barnes-Daly	Original	19	11
59	2020	Risk factors for post-intensive care syndrome: A systematic review and meta-analysis	M Lee	Review	19	19
60	2013	What follows survival of critical illness? Physical therapists' management of patients with post-intensive care syndrome	AR Bemis-Dougherty	Review	18	6

61	2017	Development and implementation of an early mobility program for mechanically ventilated pediatric patients	KA Betters	Original	17	7
62	2018	Health-related outcomes of critically ill patients with and without sepsis	K Thompson	Original	17	10
63	2017	Neurologic and functional morbidity in critically ill children with bronchiolitis*	SL Shein	Original	17	6
64	2020	Post-intensive care syndrome and COVID-19: Crisis after a crisis?	A Jaffri	Editorial/Letter	17	17
65	2018	Post-intensive care syndrome symptoms and health-related quality of life in family decision-makers of critically ill patients	AB Petrinec	Original	17	7
66	2017	Acute psychological trauma in the critically ill: Patient and family perspectives	V Dziadzko	Original	16	3
67	2017	Physical and occupational therapy utilization in a pediatric intensive care unit	LR Cui	Original	16	9
68	2018	The evolution of post intensive care syndrome	J McPeake	Editorial	16	9
69	2018	Acute physiologic stress and subsequent anxiety among family members of ICU patients	SJ Beesley	Original	15	9
70	2019	Chronic pain in critical care survivors: A narrative review	HI Kemp	Review	15	15
71	2016	Developing a diary program to minimize patient and family post-intensive care syndrome	M Locke	Review	15	4
72	2017	The ICU-Diary study: Prospective, multicenter comparative study of the impact of an ICU diary on the wellbeing of patients and families in French ICUs	M Garrouste-Orgeas	Protocol	15	10
73	2017	Pediatric delirium: Recognition, management, and outcome	SB Turkel	Review	14	2
74	2017	Six-month morbidity and mortality among intensive care unit patients receiving life-sustaining therapy: A prospective cohort study	ME Detsky	Original	14	8
75	2016	The intensive care unit experience: Psychological impact on family members of patients with and without traumatic brain injury	AM Warren	Original	14	5
76	2018	Aging and post-intensive care syndrome: A critical need for geriatric psychiatry	S Wang	Review	13	8
77	2017	Follow-up of cardiac arrest survivors: Why, how, and when? A practical approach	G Lilja	Review	13	7
78	2019	Impact of post-traumatic stress symptoms on the health-related quality of life in a cohort study with chronically critically ill patients and their partners: Age matters	GB Wintermann	Original	13	13

79	2015	The postintensive care syndrome of survivors of critical illness and their families	A Wolters	Original	13	2
80	2018	Cognitive function 3 and 12 months after ICU discharge: A prospective cohort study	S Estrup	Original	12	8
81	2017	Cognitive impairment and psychological distress at discharge from intensive care unit	CR Chung	Original	12	6
82	2019	Determinants of self-reported unacceptable outcome of intensive care treatment 1 year after discharge	MC Kerckhoffs	Original	12	12
83	2018	Development and validation of an abbreviated questionnaire to easily measure cognitive failure in ICU survivors: A multicenter study	A Wassenaar	Original	12	9
84	2018	Embracing the new vulnerable self: A grounded theory approach on critical care survivors' post-intensive care syndrome	J Kang	Original	12	6
85	2016	Natural language processing to assess documentation of features of critical illness in discharge documents of acute respiratory distress syndrome survivors	GE Weissman	Original	12	4
86	2019	Nonpharmacologic interventions to prevent or mitigate adverse long-term outcomes among ICU survivors: A systematic review and meta-analysis*	WW Geense	Review	12	12
87	2020	Patients suffering from psychological impairments following critical illness are in need of information	JH Vlake	Original	12	12
88	2020	A core outcome set for pediatric critical care*	EL Fink	Original	11	13
89	2015	Describing and measuring recovery and rehabilitation after critical illness	B Connolly	Review	11	4
90	2017	Early rehabilitation for the prevention of postintensive care syndrome in critically ill patients: A study protocol for a systematic review and metaanalysis	Y Kondo	Review	11	6
91	2017	Implementing family-centered care through facilitated sensemaking	JE Davidson	Review	11	3
92	2015	Postintensive care syndrome and the role of a follow-up clinic	JL Stollings	Review	11	1
93	2019	Psychocognitive sequelae of critical illness and correlation with 3 months follow up	LV Karnatovskaia	Original	11	11
94	2019	Validation of a new clinical tool for post-intensive care syndrome	S Wang	Original	11	11

95	2021	A clinic blueprint for post-coronavirus disease 2019 recovery learning from the past, looking to the future	DD Lutchmansingh	Review	10	10
96	2019	Chronic pain in intensive care unit survivors: Incidence, characteristics and side-effects up to one-year post-discharge	H Devine	Original	10	10
97	2019	Impact of critical illness on resource utilization: A comparison of use in the year before and after ICU admission*	EL Hirshberg	Original	10	10
98	2017	Innovation and technology: Electronic intensive care unit diaries	EA Scruth	Review	10	3
99	2017	Measuring outcomes of an intensive care unit family diary program	TG Huynh	Original	10	4
100	2016	The effect of augmenting early nutritional energy delivery on quality of life and employment status one year after ICU admission	DB Reid	Original	10	3
